# Multiple Machine Learning Approaches Based on Postoperative Prediction of Pulmonary Complications in Patients With Emergency Cerebral Hemorrhage Surgery

**DOI:** 10.3389/fsurg.2021.797872

**Published:** 2022-01-18

**Authors:** Xiaolei Jing, Xueqi Wang, Hongxia Zhuang, Xiang Fang, Hao Xu

**Affiliations:** ^1^Division of Life Sciences and Medicine, Department of Neurosurgery, The First Affiliated Hospital of USTC, University of Science and Technology of China, Hefei, China; ^2^Division of Life Sciences and Medicine, Department of Neurology, The First Affiliated Hospital of USTC, University of Science and Technology of China, Hefei, China

**Keywords:** machine learning, postoperative, postoperative pulmonary complications, emergency, cerebral hemorrhage surgery

## Abstract

**Objective:**

This study aimed to create a prediction model of postoperative pulmonary complications for the patients with emergency cerebral hemorrhage surgery.

**Methods:**

Patients with hemorrhage surgery who underwent cerebral hemorrhage surgery were included and divided into two groups: patients with or without pulmonary complications. Patient characteristics, previous history, laboratory tests, and interventions were collected. Univariate and multivariate logistic regressions were used to predict postoperative pulmonary infection. Multiple machine learning approaches have been used to compare their importance in predicting factors, namely K-nearest neighbor (KNN), stochastic gradient descent (SGD), support vector classification (SVC), random forest (RF), and logistics regression (LR), as they are the most successful and widely used models for clinical data.

**Results:**

Three hundred and fifty four patients with emergency cerebral hemorrhage surgery between January 1, 2017 and December 31, 2020 were included in the study. 53.7% (190/354) of the patients developed postoperative pulmonary complications (PPC). Stepwise logistic regression analysis revealed four independent predictive factors associated with pulmonary complications, including current smoker, lymphocyte count, clotting time, and ASA score. In addition, the RF model had an ideal predictive performance.

**Conclusions:**

According to our result, current smoker, lymphocyte count, clotting time, and ASA score were independent risks of pulmonary complications. Machine learning approaches can also provide more evidence in the prediction of pulmonary complications.

## Introduction

Complications after major surgery occur frequently and are an important cause of mortality and morbidity, especially when they affect the lungs (1). Indeed, one in every seven patients who develops a so-called postoperative pulmonary complication (PPC) dies before hospital discharge and patients who survive often suffered from a sustained reduction in functional status (2). Early identification of patients at risk of developing PPCs could enable the use of preventive measures as well as timely treatment.

However, the current predictive indicators are very limited in severe craniocerebral surgery, especially cerebral hemorrhage (3, 4). Patients with severe craniocerebral surgery often suffer from coma, lack of spontaneous breathing for a period of time, or need to be assisted breathing by the ventilator, and often combined with multiple severe multi-system symptoms. The incidence of PPC in emergency intracerebral hemorrhage (ICH) patients is much higher than that of conventional surgery, and the occurrence of complications often leads to poor prognosis, even directly related to patient death. However, there is a paucity of literature that investigates the deleterious effects of PPCs in neurosurgical patients, particularly in those requiring emergency ICH surgery which could face up to the highest rate of surgical complications rate. Therefore, we believe that better prediction of patients' PPC and taking preventive measures can greatly improve the prognosis of patients. In this study, the model of PPC in patients with ICH was established by multiple machine learning methods.

## Materials and Methods

The study was approved by our local institutional review board. The clinical data of patients who underwent emergency ICH surgery at a single institution during a 4-year period between January 1, 2017 and December 31, 2020 were reviewed and analyzed in a retrospective fashion. The characteristics of the patients included in this study were sex, age, education, medical history (coronary heart disease history, stroke history, hypertension history, and diabetes history), respiratory history, whether a current smoker, Glasgow coma scale (GCS), glucose, Albumin (Alb), WBC, lymphocyte count, leukocyte, RBC, platelet, clotting time, early enteral nutrition, preventive tracheotomy respirator use, operative time, anesthesia time, the blood loss, ASA classification, and craniotomy.

In accordance with past studies (1–7), these diagnoses were identified in critical care reports, radiographic reports, and/or the discharge summary. During the study period, Acute Respiratory Distress Syndrome (ARDS) was clinically diagnosed based on the American-European Consensus Conference on ARDS reported in 1994 (8). Outcome measures postoperative parameters included the presence of PPCs (defined as pulmonary edema, pneumonia, pneumothorax, pulmonary embolism, or ARDS). Patients who had developed PPCs during their hospital stay were compared to their non-PPC counterparts.

Statistical analysis using Student's *t*-test and one-way ANOVA was performed to determine characteristics that were statistically significantly different between the two groups. Pearson correlation analysis was performed for the risk factors and variables with *P* < 0.05 were deemed to have statistically significant associations. Variables with *P* < 0.05, as determined by univariate analysis, were included for multivariate analysis. Multivariate logistic regression analysis was employed to identify independent predictors of unfavorable outcomes.

A method that combines automatic algorithms and artificial selection aimed at dimension reduction was used for feature extraction from thousands of variables in this analysis. All features were selected by clinicians based on their experience in diagnosis before automatic analysis. The random forest algorithm was used for final extraction. According to the descending order of importance, the feature score higher than 0.0005 was selected for final analysis. Multiple algorithms were chosen to improve the probability of good discrimination performance. This study used the following classifiers: K-nearest neighbor (KNN), stochastic gradient descent (SGD), support vector classification (SVC), random forest (RF), and logistics regression (LR).

**Figure 1:**
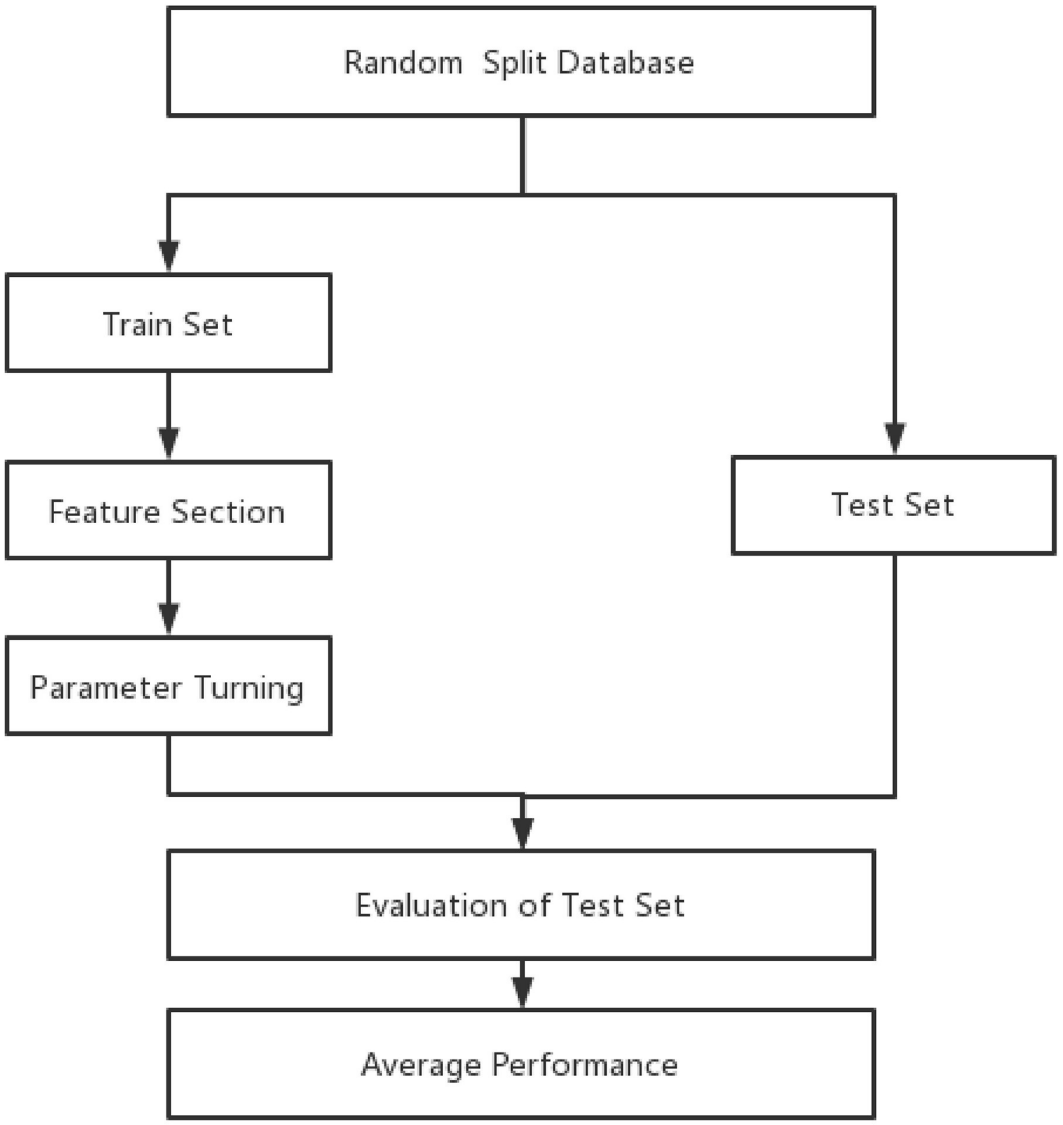
Forest plot of predictors for pulmonary.

The whole data samples were randomly split into training and test sets according to a division of 7:3. Optimal features and hyperparameters combinations for the model were determined on the training set. Furthermore, 5-fold cross-validation (23) was used in the process of feature selection and hyperparameters (Figure 1).

The important indicators of the machine learning model include precision and recall. Precision refers to the actual positive samples among all predicted positive samples. The formula is as follows: Precision = TP/(FP + TP). Recall refers to the probability of being predicted to be a positive sample in all samples. Its formula is as follows: Recall rate = TP/(TP+FN). To consider the two factors, F1 score were calculated as F1 = 2 precision-recall rate/(precision + recall rate).

To assess the discriminative performance of this risk score in both the development and validation subsamples, we used the c-statistic, which was also displayed graphically as the area under the receiver operating characteristic (ROC) curve. An area under the ROC curve (AUC) of 0.5 indicates no discrimination, whereas an AUC of 1 indicates perfect discrimination.

The model was subsequently tested on the independent test set, which had not been seen by the model during the training process so as to avoid overfitting. To avoid bias due to the random split of the training and test sets, the above procedures were repeated 10 times, and the performance of different models was compared. The comparison of different models' performance in the 10 repeats was examined by Wilcoxon signed ranks test as suggested by a previous study (9, 10). All continuous variables were normalized to the range of 0 to 1. Categorical variables were transformed into binary variables using one-hot encoding. Besides commonly used metrics such as AUC, we also reported results of the areas under the precision-recall curve, which is more informative on the imbalanced dataset. The four machine learning models were also compared.

## Results

The study included 354 patients with emergency cerebral hemorrhage surgery between January 1, 2017, and December 31, 2020. Furthermore, 53.7% (190/354) of the patients developed PPC during hospitalization. The mean age was 55.79 ± 14.31 years and the sex ratio was 71.1% in the PPC group; while the mean age was 54.77 ± 54.77 ± 18.49 years and the sex ratio was 79.9% in the non-PPC group (*P* > 0.05) (Table 1). Univariate analysis showed that there were statistically significant differences in the current smoker, ASA classification, hypertension, glucose, Alb (g/dL), WBC, leukocyte, RBC, clotting time, preventive tracheotomy, respirator use, operative time, anesthesia time, blood loss, and craniotomy between the two groups (*P* < 0.05), as shown in Table 1.

**Table 1:** Results of univariate analysis for all feature variables.

		**With PPC**	**Without PPC**	***p***	**Odds ratio**	**95% CI**
		**(*n* = 190)**	**(*n* = 164)**			
Sex		135 (71.1)	131 (79.9)	0.05	1.124	0.998–1.266
Age		55.79 ± 14.31	54.77 ± 18.49	0.55	1.004	0.991–1.017
Education (More than a high school)		28 (14.7)	20 (12.2)	0.64	1.083	0.773–1.518
Current smoker		60 (31.6)	30 (18.3)	0.004	1.194	1.058–1.347
GCS		61 (32.1)	50 (30.5)	0.74	1.024	0.889–1.179
ASA classification (≥3)		126 (66.3)	52 (31.7)	0.00	2.575	1.721–3.854
Previous history	CHD	6 (3.2)	10 (6.1)	0.18	1.931	0.717–5.198
	Stroke	26 (13.7)	28 (17.1)	0.38	1.248	0.763–2.039
	Hypertension	120 (63.2)	81 (49.4)	0.01	1.757	1.148–2.687
	Diabetes	22 (11.6)	19 (11.6)	0.99	1.001	0.562–1.782
	Pneumonia	14 (7.4)	8 (4.9)	0.33	1.027	0.974–1.083
Laboratory Test	Glucose	8.62 ± 3.40	7.14 ± 2.91	0.00	1.183	1.092–1.282
	Alb (g/dL)	36.52 ± 6.61	39.75 ± 5.43	0.00	0.914	0.881–0.951
	WBC (1,000/Cumm)	11.13 ± 9.81	5.32 ± 4.89	0.01	1.054	1.009–1.100
	LYM	1.75 ± 3.04	1.39 ± 1.65	0.18	1.066	0.969–1.172
	Leukocyte	10.46 ± 4.87	7.89 ± 4.47	0.00	1.128	1.074–1.185
	RBC	3.83 ± 0.71	4.18 ± 0.66	0.00	0.459	0.327–0.645
	Platelet (1,000/Cumm)	171.89 ± 70.89	186.50 ± 69.93	0.05	0.997	0.994–1.001
	Clotting time	20.55 ± 8.91	24.96 ± 10.12	0.00	0.953	0.931–0.975
Intervention	EEN	103 (54.2)	90 (54.9)	0.90	0.985	0.784–1.239
	Preventive tracheotomy	93 (48.9)	27 (16.5)	0.00	1.636	1.401–1.910
	Respirator use	59 (31.1)	30 (18.3)	0.01	2.012	1.219–3.321
	Operative time (minutes)	195.57 ± 93.03	148.59 ± 82.22	0.00	1.006	1.004–1.009
	Anesthesia time (minutes)	242.42 ± 106.17	181.08 ± 93.53	0	1.005	1.003–1.008
	Blood lose (ml)	180.42 ± 140.36	120.15 ± 127.11	0	1.004	1.002–1.005
	Craniotomy	42 (22.1)	66 (40.2)	0	0.421	0.265–0.670

**Figure 2:**
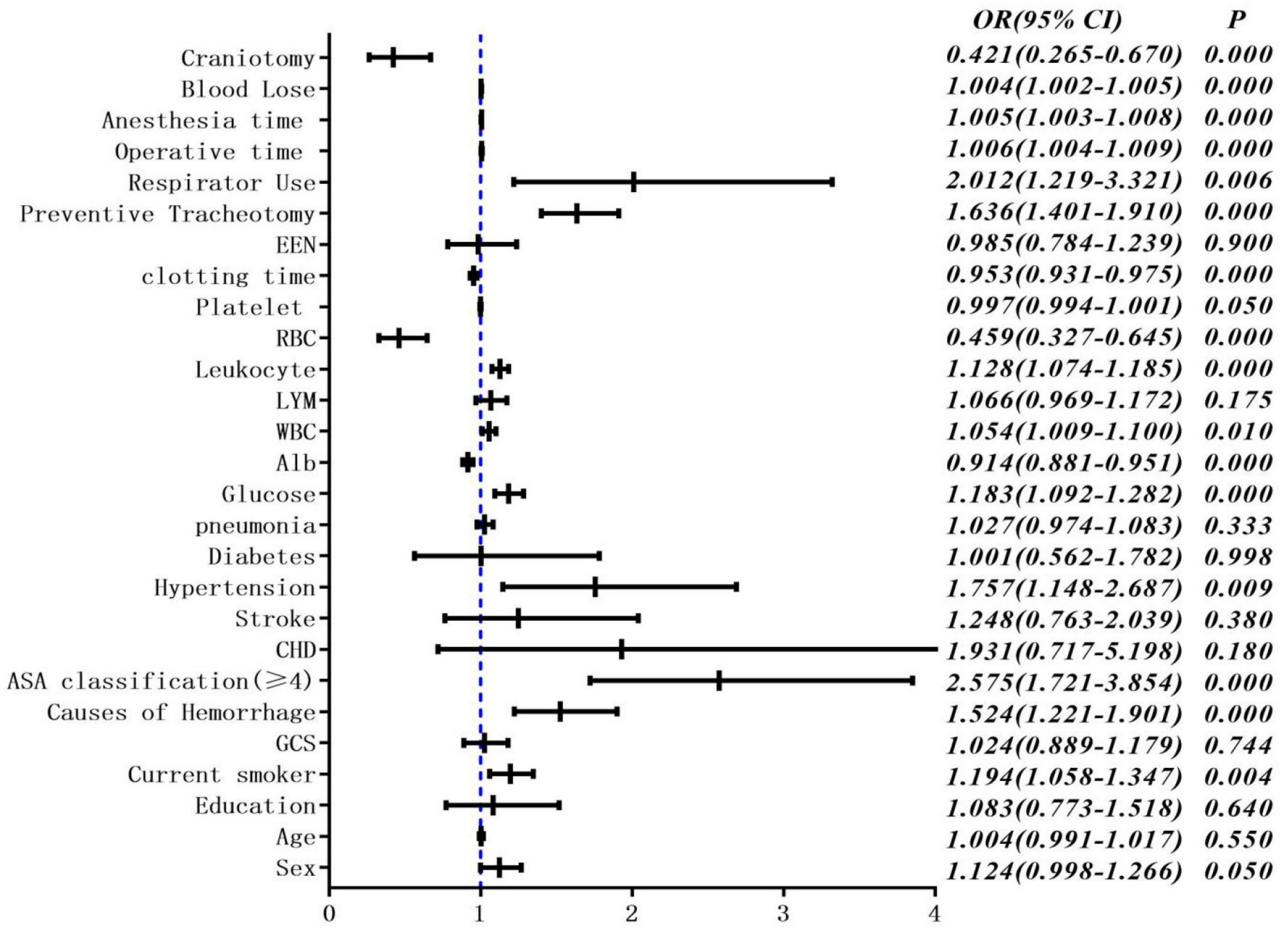
Multivariate unconditional logistic regression analysis and forest map of postoperative pulmonary complications.

The occurrence of PPC was taken as the dependent variable, and statistically significant factors in univariate analysis were taken as independent variables. Logistic regression analysis was performed. Variables were screened by stepwise method (the model inclusion level was 0.05 and the exclusion level was 0.1). The results showed that the chi-square test of likelihood ratio suggested that the regression model had statistical significance (*P* < 0.05). Current smoker, lymphocyte count, clotting time, and ASA classification were all independent influencing factors for the occurrence of pulmonary complications (Table 2, Figure 2).

**Table 2:** Multivariate unconditional Logistic regression analysis of postoperative pulmonary complications.

	**β**	**SE**	**woldX2**	***P***	**OR**	**95% CI**
Constant	−2.129	0.689	9.56	0.002	0.119	
Current smoker	0.702	0.29	5.874	0.015	2.018	1.144~3.56
Leukocyte	0.074	0.026	7.991	0.005	1.077	1.023~1.134
Coltting time	−0.056	0.014	16.002	0	0.946	0.92~0.972
ASA	1.116	0.179	39.047	0	3.052	2.151~4.331

**Figure 3:**
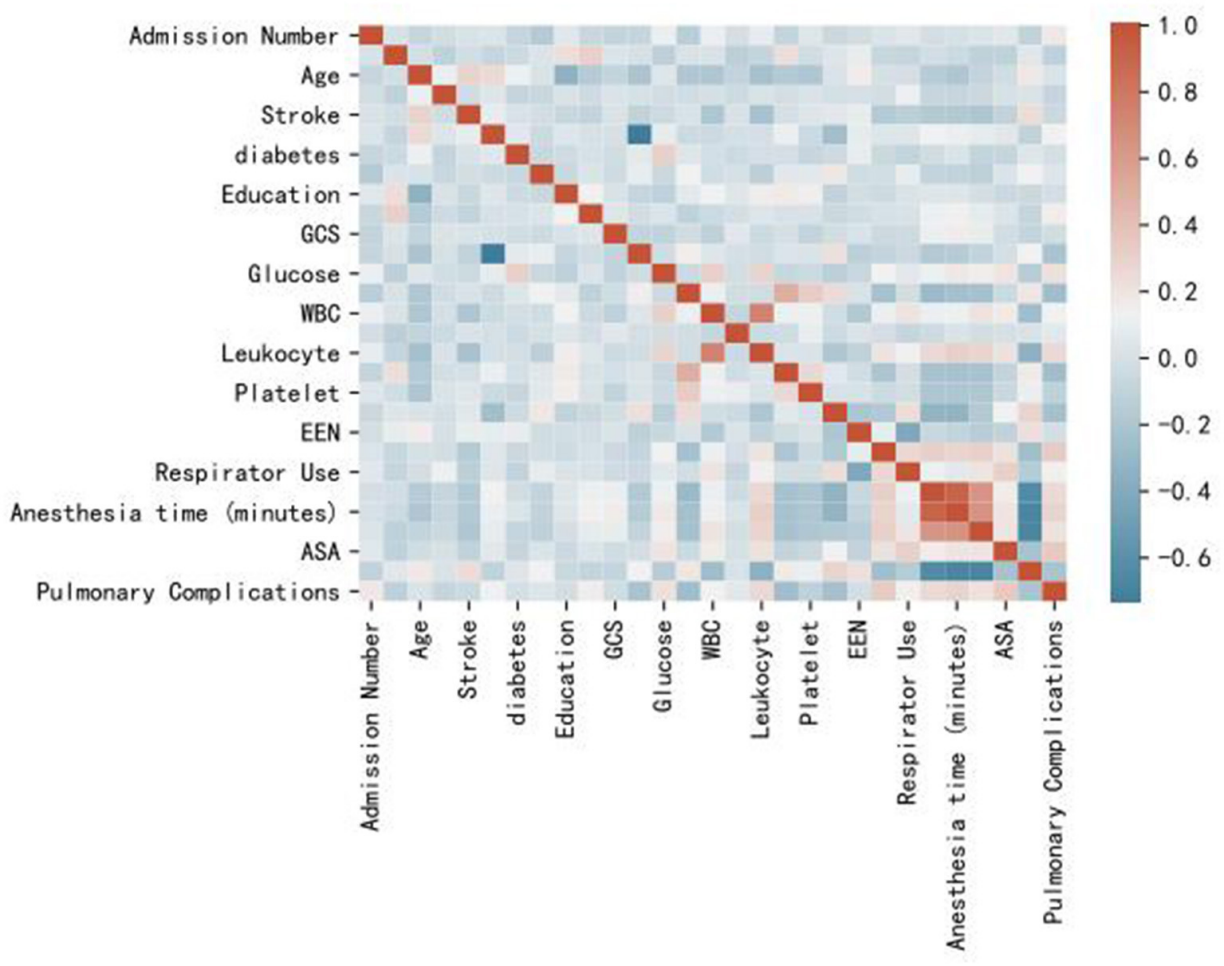
Heat map of correlation analysis results indicates the the risk factors association with PPC.

**Figure 4:**
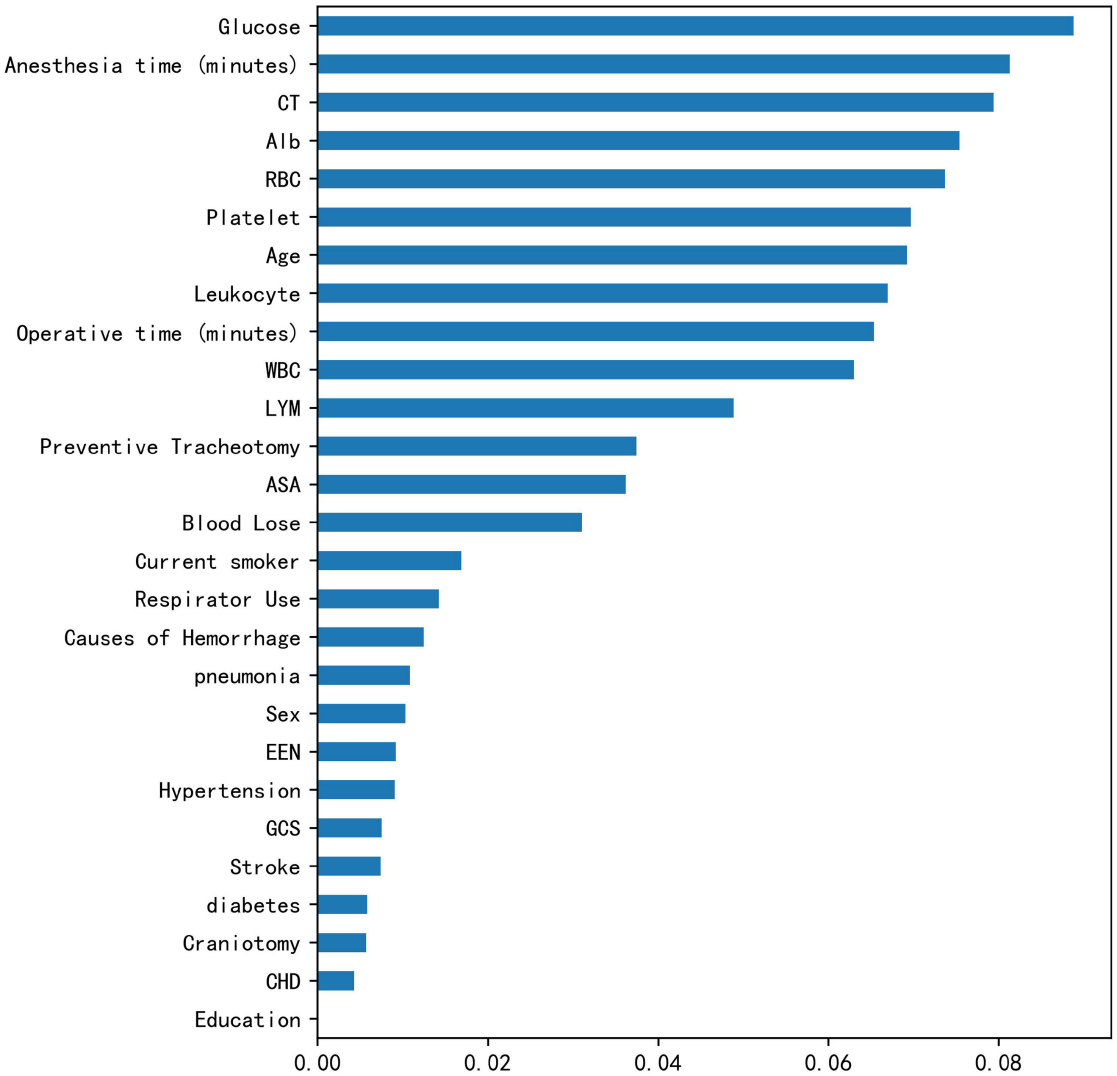
Rank of feature importance of PPC in RF model.

**Figure 5:**
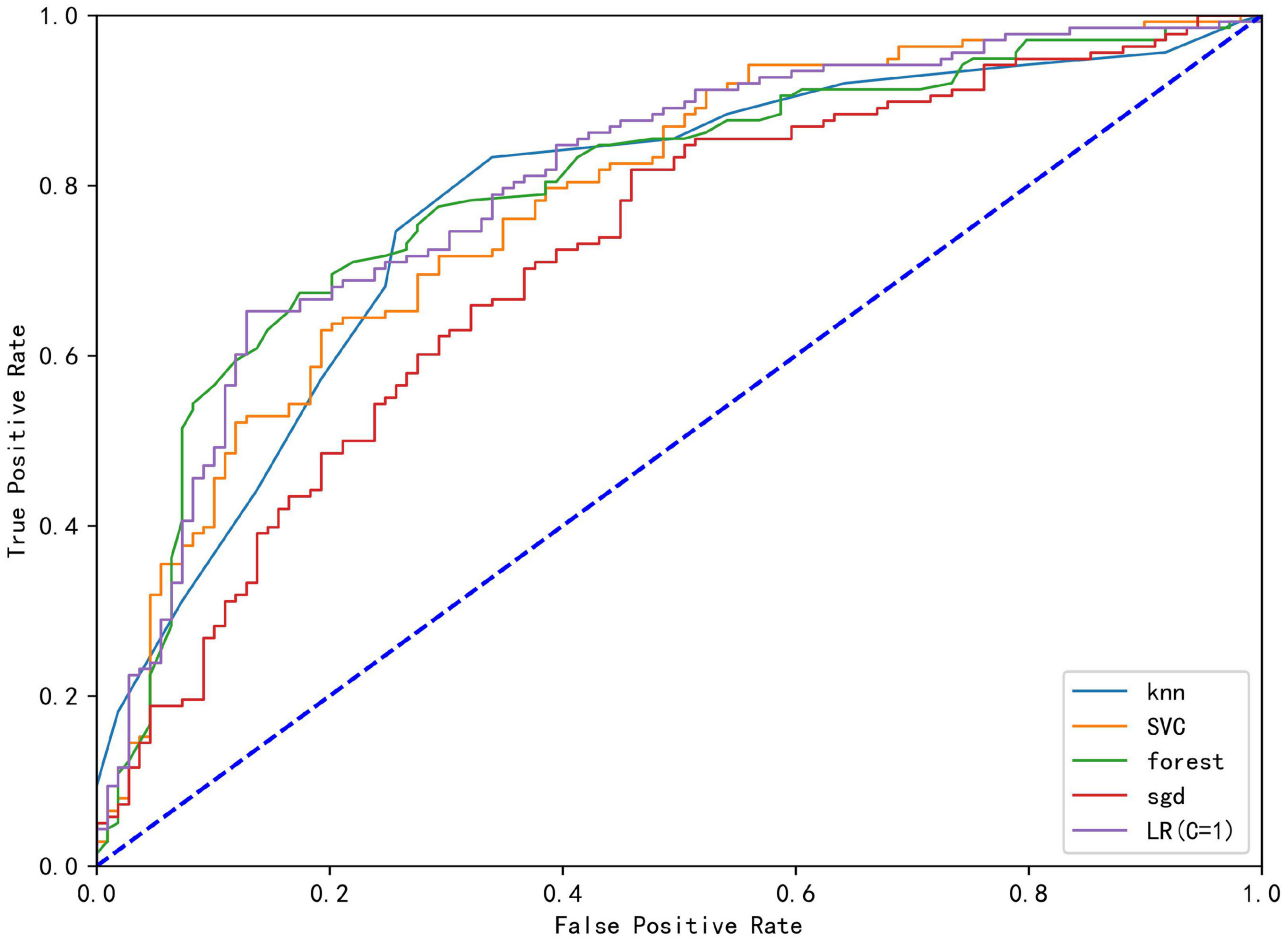
The ROC curve analysis of the four derived models (KNN), Stochastic Gradient Descent (SGD), Support Vector Classification (SVC), Random Forest (RF), Stochastic Gradient Descent (SGD) and logistics regression (LR).

In the correlation analysis, we could see that glucose (0.225705), operative time (0.257506), leukocyte (0.264244), anesthesia time (0.291870), preventive tracheotomy (0.342191), ASA (0.345156) was closely correlated with PPC (Figure 3). In the RF model, we observed the importance of features, and the top five are glucose, lymphocyte counterpoint, clotting time, anesthesia time, and Alb (Figure 4). The ROC curves of the five derived models are plotted in Figure 5. The model achieved the highest AUC of 0.653, followed by the LR model of 0.774194. SGD (0.712871) model showed a relatively poor result in the ROC curve. When we observed f1, RF also performs relatively well, especially the f1 value of 0.69 in the test set (Table 3).

**Table 3:** Performance comparison of machine learning model.

**Model**	**Linear SVC**	**KNN**	**Random forest**	**SGD**	**LR (C = 1)**
Train set precision	0.734848	0.78626	0.756757	0.716535	0.765957
Train set recall	0.702899	0.746377	0.811594	0.65942	0.782609
Train set f1	0.718519	0.765799	0.783217	0.686792	0.774194
Train set ROC area	0.780282	0.776393	0.794475	0.712871	0.78693
Test set percision	0.666667	0.607143	0.655172	0.634146	0.661017
Test set recall	0.730769	0.653846	0.730769	0.500000	0.750000
Test set f1	0.697248	0.62963	0.690909	0.55914	0.702703
Test set ROC area	0.683916	0.616783	0.652797	0.637063	0.665734

## Discussion

Postoperative pulmonary complications (PPC) are a well-described cause of post-surgical detrimental outcomes, including intensive care unit admission, prolonged admissions, perioperative mortality, and increased hospital expenditures in patients who underwent surgery. Moreover, the complication rate of neurosurgery is naturally high. Previous studies have shown that pulmonary complications occur between 1.3 and 22%, depending on the different types of neurosurgery (1–4, 11). Now there are some predictors of pulmonary complications, such as the “Assess Respiratory Risk in Surgical Patients in Catalonia” (ARISCAT) risk score, the “Surgical Lung Injury Prediction” (SLIP) model, and LAS VEGAS risk score which are two well-established prediction scores used for the identification of patients at risk of developing PPC or ARDS, respectively (12, 13). But these indicators are inapplicable to neurosurgery in clinical practice.

In this study, the incidence of PPC reached 53.7%. Since all our patients were in emergent and severe conditions comparative, the incidence of PPC tended to be higher. Some studies have shown that the mortality rate of patients undergoing decompressive craniectomy is as high as 40.9%. In this study, traditional logistic reviews identified some independent risk factors by univariate and multivariate regression analyses. In particular, current smoker, lymphocyte count, clotting time, and ASA classification were independent risk factors for PPC. It was basically consistent with the results of previous studies (14–20). However, we noticed that some important risk factors reported in previous literature, such as patients' blood glucose level and operation time, had not reached the multivariate regression inclusion criteria in our study. We thought it might be due to insufficient sample size, or our review of pulmonary complications was relatively broad.

In terms of preventing PPC, some of these risk factors are controllable and some are not. Careful timing of surgery, smoking reduction, regulation of blood glucose, and preventive use of antibiotics could minimize complications. However, most of the thrombo-embolic events are often unpredictable and unpreventable, and low molecular weight heparin in the context of protocols for thromboprophylaxis could also be a beneficial attempt (21).

In recent years, machine learning has been used to predict the prognosis of various neurological diseases with remarkable results (22–27). RF is an ensemble learner composed of the decision tree, which also highlights the importance of each indicator. In this study, RF cast light on the importance of blood glucose indicators. In contrast, the *p*-value of blood glucose is on the margin of 0.05, so that it could be missing in the analysis. Comprehensively, the LR model performed better overall, including in the training set and test set. In particular, the test set performance remained stable, far outperforming other machine learning methods. As a most popular machine learning algorithm, RF provides accurate results without exhaustive hyper-parameter tuning and can be applied to both regression and classification problems, when the number of potential explanatory variables is far more than the observed values. In addition, all the other five models showed moderate classification ability (AUC ranging from 0.6 to 0.71). The current study could be considered as a novel exploration of the modified machine learning approach for PPC. In particular, the machine learning model can find some potential risk factors, such as the blood glucose index in this paper that could not be found in previous studies due to different learning models, especially in the case of limited sample size.

Although machine learning models are powerful, they are often more complex, which makes them difficult to understand like a: “black box” (28). Therefore, the interpretation of machine learning results particularly depends on the experience of clinicians, especially for the prediction of complications, in order to identify high-risk patients and adapt treatment plans as early as possible, so as to reduce the incidence of complications and improve the prognosis of patients. According to different situations, we can adjust the recall rate appropriately to avoid missing high-risk patients, and the requirement for precision can be relaxed, because once missing patients with pulmonary complications, it may cause serious consequences. For example, in this study, RF model with the parameters C = 1, precision = 0.76, recall rate = 0.82 is a relatively good prediction model.

This study has several limitations. First, the diagnosis of PPC relied on the attending physicians' evaluation in this retrospective study; therefore, the potential of either underestimation or overestimation of the actual incidence of PPC could not be avoided. Our inclusion indicators were relatively loose, and although some patients were diagnosed with pulmonary edema, they did not need special intervention. Second, the definition of PPC was based on radiology evidence rather than etiological results. Another limitation of the study is that the diagnosis of PPC was occasionally a clinical one and that there was no clear source of infection. At the same time, our study was a retrospective analysis, and the number of specimens was relatively low considering the large amount required in machine learning analysis. Fortunately, we have adopted a variety of machine learning models to analyze and process the data to minimize the omission of important indicators.

Finally, in this study, the prediction models of pulmonary complications in patients with severe emergency ICH were established. Compared with traditional statistical methods, the machine learning model was more comprehensive and flexible, providing new ideas for the prediction model of pulmonary complications in the future.

## Data Availability Statement

The original contributions presented in the study are included in the article/supplementary material, further inquiries can be directed to the corresponding authors.

## Ethics Statement

The studies involving human participants were reviewed and approved by the Ethics Committee of the First Affiliated Hospital of University of Science and Technology of China (Hefei, China). Written informed consent from the patients/participants or patients/participants legal guardian/next of kin was not required to participate in this study in accordance with the national legislation and the institutional requirements.

## Author Contributions

All authors listed have made a substantial, direct, and intellectual contribution to the work and approved it for publication.

## Funding

The present study was supported by the Fundamental Research Funds for the Central Universities (Grant no. WK9110000126).

## Conflict of Interest

The authors declare that the research was conducted in the absence of any commercial or financial relationships that could be construed as a potential conflict of interest.

## Publisher's Note

All claims expressed in this article are solely those of the authors and do not necessarily represent those of their affiliated organizations, or those of the publisher, the editors and the reviewers. Any product that may be evaluated in this article, or claim that may be made by its manufacturer, is not guaranteed or endorsed by the publisher.

## References

[1] Fernandez-Bustamante A Frendl G Sprung J Kor DJ Subramaniam B Martinez Ruiz R. Postoperative pulmonary complications, early mortality, and hospital stay following noncardiothoracic surgery: a multicenter study by the perioperative research network investigators. JAMA Surg. (2017) 152:157–66. 10.1001/jamasurg.2016.4065 27829093 PMC5334462

[2] Miskovic A Lumb AB. Postoperative pulmonary complications. Br J Anaesth. (2017) 118:317–34. 10.1093/bja/aex002 28186222

[3] Canet J Gallart L Gomar C Paluzie G Vallès J Castillo J. Prediction of postoperative pulmonary complications in a population-based surgical cohort. Anesthesiology. (2010) 113:1338–50. 10.1097/ALN.0b013e3181fc6e0a 21045639

[4] Sabaté S Mazo V Canet J. Predicting postoperative pulmonary complications: implications for outcomes and costs. Curr Opin Anaesthesiol. (2014) 27:201–9. 10.1097/ACO.0000000000000045 24419159

[5] Cai Y-H Wang H-T Zhou J-X. Perioperative predictors of extubation failure and the effect on clinical outcome after infratentorial craniotomy. Med Sci Monit. (2016) 22:2431–8. 10.12659/MSM.899780 27404044 PMC4944551

[6] Chu H Dang B-W. Risk factors of postoperative pulmonary complications following elective craniotomy for patients with tumors of the brainstem or adjacent to the brainstem. Oncol Lett. (2014) 8:1477–81. 10.3892/ol.2014.2374 25202352 PMC4156239

[7] Su Z Liu S Oto J. Effects of positive endexpiratory pressure on the risk of postoperative pulmonary complications in patients undergoing elective craniotomy. World Neurosurg. (2018) 112:e39–49. 10.1016/j.wneu.2017.12.014 29253690

[8] Bernard GR Artigas A Brigham KL Carlet J Falke K Hudson L. The American-European Consensus Conference on ARDS. Definitions, mechanisms, relevant outcomes, and clinical trial coordination. Am J Respir Crit Care Med. (1994) 149(3 Pt 1):818–24. 10.1164/ajrccm.149.3.7509706 7509706

[9] Flexman AM Merriman B Griesdale DE Mayson K Choi PT Ryerson CJ. Infratentorial neurosurgery is an independent risk factor for respiratory failure and death in patients undergoing intracranial tumor resection. J Neurosurg Anesthesiol. (2014) 26:198–204. 10.1097/ANA.0b013e3182a43ed8 23933960

[10] Oh T Safaee M Sun MZ Garcia RM McDermott MW Parsa AT. Surgical risk factors for post-operative pneumonia following meningioma resection. Clin Neurol Neurosurg. (2014) 118:76–9. 10.1016/j.clineuro.2013.12.017 24529234

[11] Cai YH Zeng H-Y Shi Z-H. Factors influencing delayed extubation after infratentorial craniotomy for tumour resection: a prospective cohort study of 800 patients in a Chinese neurosurgical centre. J Int Med Res. (2013) 41:208–17. 10.1177/0300060513475964 23569147

[12] Kor DJ Warner DO Alsara A Fernández-Pérez ER Malinchoc M Kashyap R. Derivation and diagnostic precision of the surgical lung injury prediction model. Anesthesiology. (2011) 115:117–28. 10.1097/ALN.0b013e31821b5839 21694510 PMC3986041

[13] Kor DJ Lingineni RK Gajic O Park PK Blum JM Hou PC. Predicting risk of postoperative lung injury in high-risk surgical patients: a multicenter cohort study. Anesthesiology. (2014) 120:1168–81. 10.1097/ALN.0000000000000216 24755786 PMC3999474

[14] Di Battista AP Rizoli SB Lejnieks B Min A Shiu MY Peng HT. Sympathoadrenal activation is associated with acute traumatic coagulopathy and endotheliopathy in isolated brain injury. Shock. (2016) 46(3 suppl 1):96–103. 10.1097/SHK.0000000000000642 27206278 PMC4978599

[15] Nakae R Yokobori S Takayama Y Kanaya T Fujiki Y Igarashi Y. A retrospective study of the effect of fibrinogen levels during fresh frozen plasma transfusion in patients with traumatic brain injury. Acta Neurochir. (2019) 161:1943–53. 10.1007/s00701-019-04010-3 31309303

[16] Engström M Romner B Schalén W Reinstrup P. Thrombocytopenia predicts progressive hemorrhage after head trauma. J Neurotrauma. (2005) 22:291–6. 10.1089/neu.2005.22.291 15716634

[17] Flint AC Manley GT Gean AD Hemphill JC 3rd Rosenthal G. Post-operative expansion of hemorrhagic contusions after unilateral decompressive hemicraniectomy in severe traumatic brain injury. J Neurotrauma. (2008) 25:503–12. 10.1089/neu.2007.0442 18346002

[18] Marshall LF Marshall SB Klauber MR Van Berkum Clark M Eisenberg H Jane JA. The diagnosis of head injury requires a classification based on computed axial tomography. J Neurotrauma. (1992) 9:S287–92. 1588618

[19] Jacobs B Beems T Van der Vliet TM DiazArrastia RR Borm GF Vos PE. Computed tomography and outcome in moderate and severe traumatic brain injury: hematoma volume and midline shift revisited. J Neurotrauma. (2011) 28:203–15. 10.1089/neu.2010.1558 21294647

[20] Kuo JR Lo CJ Lu CL Chio CC Wang CC Lin KC. Prognostic predictors of outcome in an operative series in traumatic brain injury patients. J Formos Med Assoc. (2011) 110:258–64. 10.1016/S0929-6646(11)60038-7 21540008

[21] Chibbaro S Cebula H Todeschi J Fricia M Vigouroux D Abid H. Evolution of prophylaxis protocols for venous thromboembolism in neurosurgery: results from a prospective comparative study on low-molecular-weight heparin, elastic stockings, and intermittent pneumatic compression devices. World Neurosurg. (2018) 109:e510–6. 10.1016/j.wneu.2017.10.012 29033376

[22] Bhandari A Koppen J Agzarian M. Convolutional neural networks for brain tumour segmentation. Insights Imaging. (2020) 11:77. 10.1186/s13244-020-00869-4 32514649 PMC7280397

[23] Raj R Luostarinen T Pursiainen E Posti JP Takala RSK Bendel S. Machine learning-based dynamic mortality prediction after traumatic brain injury. Sci Rep. (2019) 9:17672. 10.1038/s41598-019-53889-6 31776366 PMC6881446

[24] Haveman ME Van Putten MJAM Hom HW Eertman Meyer CJ Beishuizen A Tjepkema-Cloostermans MC. Predicting outcome in patients with moderate to severe traumatic brain injury using electroencephalography. Crit Care. (2019) 23:401. 10.1186/s13054-019-2656-6 31829226 PMC6907281

[25] Matsuo K Aihara H Nakai T Morishita A Tohma Y Kohmura E. Machine learning to predict in-hospital morbidity and mortality after traumatic brain injury. J Neurotrauma. (2020) 37:202–10. 10.1089/neu.2018.6276 31359814

[26] Hale AT Stonko DP Brown A Lim J Voce DJ Gannon SR. Machinelearning analysis outperforms conventional statistical models and CT classification systems in predicting 6-month outcomes in pediatric patients sustaining traumatic brain injury. Neurosurg Focus. (2018) 45:E2. 10.3171/2018.8.FOCUS17773 30453455

[27] Senders JT Staples PC Karhade AV Zaki MM Gormley WB Broekman MLD. Machine learning and neurosurgical outcome prediction: a systematic review. World Neurosurg. (2018) 109:476–86.e1. 10.1016/j.wneu.2017.09.149 28986230

[28] Liao P Wu H Yu T. ROC curve analysis in the presence of imperfect reference standards. Stat Biosci. (2017) 9:91–104. 10.1007/s12561-016-9159-7 28694878 PMC5501420

